# Clinical outcomes of coronal shear fractures of the distal humerus associated with olecranon fractures

**DOI:** 10.1016/j.xrrt.2025.100654

**Published:** 2025-12-24

**Authors:** Momosuke Shoda, Takahiro Yamazaki, Yusuke Matsuura, Takeru Ohara, Hiromasa Wakita, Seiji Ohtori

**Affiliations:** a Department of Orthopaedic Surgery, Numazu City Hospital, Numazu City, Shizuoka, Japan; b Department of Orthopaedic Surgery, Graduate School of Medicine, Chiba University, Chiba, Chiba, Japan; c Department of Orthopaedic Surgery, Funabashi Municipal Medical Center, Funabashi City, Chiba, Japan; d Department of Orthopaedic Surgery, Eastern Chiba Medical Center, Tōgane City, Japan

**Keywords:** Coronal shear fracture, Capitellum, Trochlea, Distal humerus, Olecranon fracture, Dubberley classification, Posterolateral approach, Mayo Elbow performance Score

## Abstract

**Background:**

Coronal shear fracture (CSF) of the distal humerus is rare, accounting for approximately 1% of elbow fractures; cases combined with olecranon fracture are even rarer. This study aimed to clarify the relationship between surgical methods and clinical outcomes in this uncommon injury.

**Methods:**

Between 2017 and 2025, we retrospectively reviewed 8 patients (8 elbows) with CSF associated with olecranon fracture who underwent open reduction and internal fixation in a multicenter case series and were followed for more than 6 months. Patient demographics, fracture classifications, surgical methods, postoperative complications, additional surgeries, elbow range of motion, and Mayo Elbow Performance Score at the final follow-up were analyzed descriptively.

**Results:**

The mean age was 64 years (range, 51–79 years), and the mean follow-up was 27 months (range, 10–96 months). Dubberley classification was type 2A in 2 cases, type 3A in 2, and type 3B in 4. A posterolateral extended approach was used in 6 patients and a combined lateral–posterior approach in 2. Postoperative complications included elbow release in 2 cases and ulnar neuropathy in 2. At final follow-up, the mean Mayo Elbow Performance Score was 92.5 (range, 75–100), and the mean range of motion was −21°/121.3°.

**Conclusion:**

Outcomes of CSF with concomitant olecranon fracture appeared to depend on CSF severity, and complications were more frequent than in isolated CSF. Given the small sample size and observational design, these findings should be regarded as preliminary and hypothesis-generating rather than definitive evidence favoring any particular method.

Coronal shear fracture (CSF) of the distal humerus is a rare intra-articular injury, accounting for about 1% of elbow fractures and 6% of distal humeral fractures.^4^ Because displacement can lead to functional impairment, open reduction and internal fixation (ORIF) is commonly required.^4^ The “double-arc sign” described by McKee et al^7^ is a characteristic radiographic finding suggesting extension of the fracture to the lateral trochlea. Later studies showed that injuries appearing as isolated capitellar fractures often have more complex intra-articular extensions.^8^

CSF combined with an olecranon fracture is extremely rare. Inoue and Horii (1992) and Sun et al^6^^,^^10^ proposed that direct trauma to a flexed elbow may first cause an olecranon fracture, with transmitted shear force subsequently producing capitellar and trochlear fractures.

Although ORIF is the standard treatment, the optimal strategy when an olecranon fracture coexists has not been established. Guitton et al^5^ reported poorer outcomes in Dubberley type 3 than in type 1 fractures, indicating prognosis relates to fracture type.

This study aimed to evaluate clinical outcomes of distal humeral CSF with associated olecranon fractures in a multicenter setting and to clarify the relationship between surgical techniques and outcomes.

## Materials and methods

Patients: Eight patients with CSF and concomitant olecranon fracture treated with ORIF between 2017 and 2025 at 4 institutions were included. Exclusion criteria were pediatric cases (younger than 18 years), polytrauma, and incomplete records. These 8 patients represented all cases of distal humeral CSF with concomitant ipsilateral olecranon fracture treated surgically at the 4 institutions during the study period. Although pediatric cases, polytrauma, and incomplete records were predefined as exclusion criteria, no patients in this cohort met these criteria and no eligible patients were treated nonoperatively.

Evaluation items: The primary outcome was Mayo Elbow Performance Score (MEPS) at the final follow-up. Secondary outcomes included demographics; fracture classifications (Dubberley for CSF,^3^ Colton for olecranon^2^); surgical approaches and implants; postoperative complications; additional surgeries; and final elbow range of motion (ROM).

Postoperative management: In the case treated with a temporary external fixator, the fixator was maintained for 1 month after surgery and then removed. In all cases, the elbow was immobilized in a splint for approximately 2 weeks postoperatively, after which elbow ROM exercises were initiated according to pain and wound condition. Heterotopic ossification prophylaxis was not routinely performed.

Statistical analysis: Descriptive statistics were used to calculate means and ranges.

## Results

Demographics: Eight patients (2 men, 6 women) with a mean age of 64 years (51–79 years) and a mean follow-up of 27 months (10–96 months) were included. All cases are listed in Table I. The mechanism of injury was a fall in 7 patients and a jump from the second floor in 1 patient. Among the 7 falls, 6 occurred as a direct impact onto a flexed elbow, whereas 1 patient could not clearly recall which part of the body struck the ground.

**Table I:** Patient demographics, fracture classification, surgical methods, complications, and outcomes.

Variable	Case 1	Case 2	Case 3	Case 4	Case 5	Case 6	Case 7	Case 8
Age (yr)	51	79	62	62	67	66	72	53
Sex	Male	Female	Female	Female	Female	Female	Female	Male
Follow-up (month)	10	20	24	28	96	11	15	12
Dubberly Classification	3B	3 A	2A	3 B	3A	2 A	3B	3 B
Colton Classification	G1	G2A	G1	G2A	G2A	G2A radial head Fx	G2A	G1
Incision	Posterolateral	Posterolateral	Posterolateral	Posterolateral	Two incision	Two incision	Posterolateral	Posterolateral
Fixation								
CSF	HS posterior plate	HS	HS	HS posterior plate external fixation	HS external fixation	HS	HS external fixation	HS mini-plate
Olecranon	Wiring	Wiring	Plate	Plate	Wiring	Wiring	Wiring	Wiring
Complication	Wound trouble	None	Ulnar nerve disorder	Heterotopic ossification	Ulnar nerve disorder	None	None	Screw backout
Additional operation	Wound débridemen Joint mobilization	None	Nerve transposition	Joint mobilization	None	None	None	Implant removal
Range of motion (elbow ext/flex)	ー50/110	ー10/130	0/130	ー40/85	ー20/125	ー5/130	ー30/130	ー10/130
MEPS	80	100	100	75	100	100	85	100

*MEPS*, Mayo Elbow Performance Score; *CSF*, coronal shear fracture; *HS*, headless screw.

Fracture classification: Dubberley classification: type 2A in 2, type 3A in 2, and type 3B in 4. Colton classification: G1 in 3 and G2A in 5. One patient had a concomitant radial head fracture.

Surgical methods: A posterolateral extended approach was used in 6 cases and a combined lateral–posterior approach in 2. All CSFs were fixed with headless screws; type 3B cases additionally required posterolateral plating, hinged external fixation, or anterior mini-plate augmentation. Olecranon fractures were fixed with tension-band wiring in 6 and plating in 2.

Complications and reoperations: Postoperative complications included wound dehiscence (n = 1), ulnar neuropathy (n = 2), heterotopic ossification (n = 1), and screw backout (n = 1). Four patients (50%) required reoperation: débridement (n = 1), anterior ulnar nerve transposition (n = 1), elbow release (n = 2), and screw removal (n = 1). The time from injury to surgery ranged from 3 to 10 days. The only patient who developed wound dehiscence underwent surgery 10 days after injury and was also the one injured by jumping from the second floor, which may have contributed to the soft-tissue condition and subsequent wound problem.

Final outcomes: All fractures united; no nonunion occurred. Mean ROM was −21° extension (range, −50° to 0°) and 121.3° flexion (85°–130°). Mean MEPS was 92.5 (75–100): excellent in 5, good in 2, and fair in 1. Dubberley 3 B fractures had lower mean MEPS (85). Cases treated with posterolateral plating frequently required elbow release, whereas those managed with external fixation or an anterior mini-plate tended to have better ROM and MEPS than those treated with posterolateral plating, but given the very small sample size, these differences should be interpreted with caution and cannot be considered statistically significant.

### Representative case (case 4)

**Figure 1:**
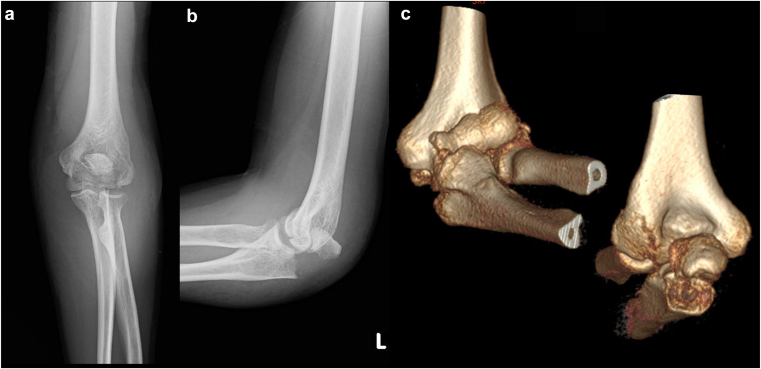
Preoperative plain radiographs and CT images. (**a**) Anteroposterior radiograph, (**b**) lateral radiograph, (**c**) 3D CT reconstruction. *CT*, computed tomography; *3D*, three-dimensional.

**Figure 2:**
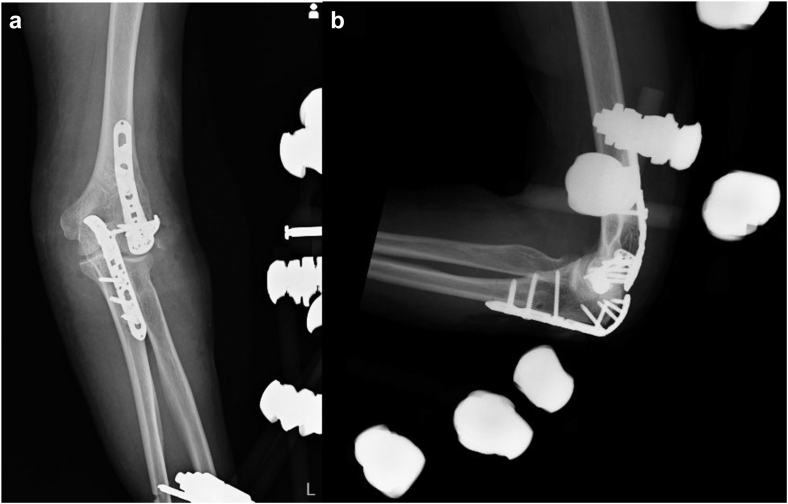
Postoperative plain radiographs. (**a**) Anteroposterior radiograph, (**b**) lateral radiograph.

**Figure 3:**
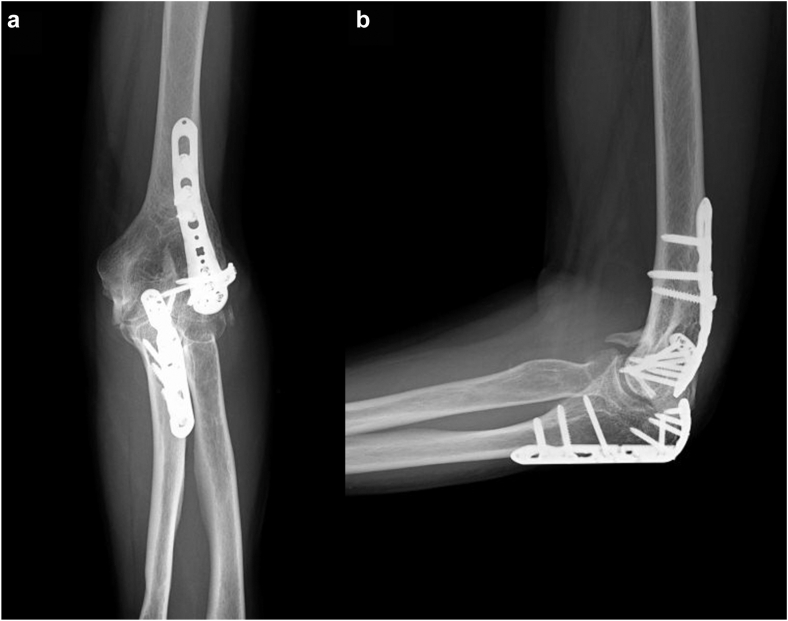
Plain radiographs before resection of heterotopic ossification. (**a**) Anteroposterior radiograph, (**b**) lateral radiograph.

**Figure 4:**
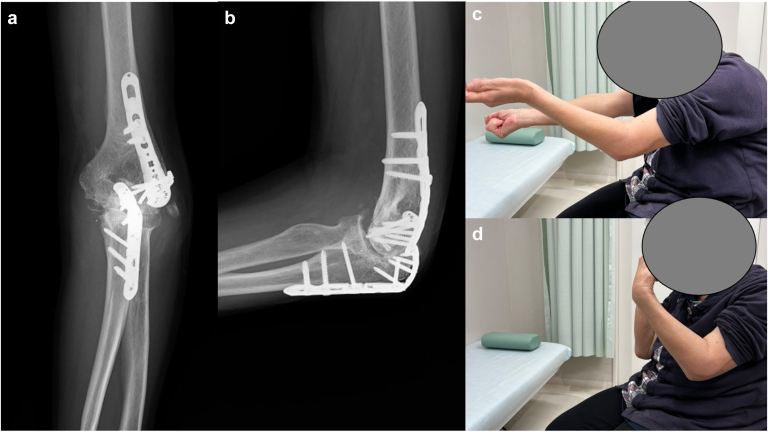
Plain radiographs and clinical photographs after resection of heterotopic ossification. (**a**) Anteroposterior radiograph, (**b**) lateral radiograph, (**c**) extension of the elbow showing −50°, (**d**) flexion of the elbow showing 85°.

A 62-year-old woman sustained an injury after a fall. Plain radiographs and CT showed a left capitellar fracture (Dubberley type 3B) with a concomitant olecranon fracture (Colton G2A) (Fig. 1). Surgery was performed 1 week after injury under general anesthesia. An arcuate skin incision was made between the olecranon and the lateral aspect of the elbow. First, the anterior joint surface was exposed to address the CSF, and the fragments were fixed with headless screws and a posterolateral plate. The olecranon was then fixed with a plate through the same incision (Fig. 2). Because trochlear fixation was felt to be insufficiently stable, a hinged external fixator was applied and removed 1 month postoperatively. An orthosis was used for 2 months postoperatively. At 6 months, severe limitation of motion due to heterotopic ossification was observed (Fig. 3), and elbow release was performed. At the final follow-up (Fig. 4), the patient was pain-free but had restricted motion with −40° extension and 85° flexion, without varus–valgus instability. The hand could not reach the face, and the MEPS was 75.

## Discussion

Injury mechanism and pathology: The typical mechanism of CSF is indirect force to the palm with the elbow in hyperextension or slight flexion. However, when an olecranon fracture coexists, the mechanism may differ. Sun et al suggested that direct trauma to a flexed elbow first causes an olecranon fracture, transmitting force to the distal humerus and resulting in CSF. Our findings support this mechanism.

Surgical approach and exposure: Multiple approaches to CSF have been described, including anterolateral, lateral, and posterior. In cases with olecranon fractures, a posterior approach is frequently preferred. Guitton et al described using an olecranon osteotomy in 3 cases and an existing olecranon fracture in 2 for exposure, which aligns with using the fracture site as a “window.” In our series, the posterolateral extended approach provided 3 advantages: (1) exposure through the olecranon fracture, (2) good visualization of the anterior articular surface, and (3) the ability to place both a posterolateral plate and olecranon fixation through the same exposure.

Fixation methods: Headless compression screws are widely used for fixation of distal humeral CSF.^4^ In the present series, headless screws were employed in all cases and provided stable fixation. In Dubberley 3B patterns with posterior cortical comminution, adjunct support with a posterolateral plate or a hinged external fixator has been recommended.^3^ In our cohort, however, cases treated with a posterolateral plate demonstrated poorer ROM and lower MEPS, suggesting that posterolateral plating should be approached with caution in CSF combined with an olecranon fracture. Because posterior wall comminution can leave residual instability when headless screws are used alone, supplemental stabilization with external fixation or an anterior mini-buttress plate merits consideration. Song et al^9^ described adding a small anterior plate along the anterior articular margin as a buttress in unstable or comminuted CSF—particularly Dubberley B/3B—with favorable clinical outcomes. Consistent with that rationale, the Dubberley 3B case in our series treated with an anterior plate achieved good ROM and MEPS.

For the olecranon component, tension-band wiring is recommended for simple fracture patterns, whereas plating is preferred for comminuted fractures or fracture-dislocations.^2^ In our series, fixation methods were selected according to fracture morphology, and no meaningful differences in outcome were attributable to the choice of olecranon fixation.

Postoperative complications: Half of our patients required reoperation, a higher proportion than in isolated CSF. Guitton et al reported that 67% required additional surgery, mostly implant removal. Two of our Dubberley 3B cases treated with posterolateral plates required elbow release, reflecting greater invasiveness and a higher risk of stiffness. Ulnar neuropathy occurred in 2 cases, improving after anterior transposition in 1. Cho et al^1^ reported extremely high complication rates (94%) in trans-olecranon distal humerus fractures, supporting the notion that combined distal humerus and olecranon fractures have poor outcomes.

Clinical outcomes and prognosis: At final follow-up, the mean MEPS was 92.5, similar to long-term results reported by Guitton et al. However, Dubberley 3B cases showed lower scores (mean 85), consistent with previous studies. ROM outcomes (mean −21°/121.3°) were comparable to Guitton's reported 119°, indicating generally satisfactory motion recovery except in severe cases. In particular, all fair or poor outcomes in our series occurred in Dubberley type 3 fractures, which support previous reports that more severe CSF patterns are associated with worse prognosis.

Limitations: This study is limited by its small sample size and retrospective design. As a multicenter case series of only 8 patients, it is clearly underpowered to detect statistically significant differences between surgical techniques, and all findings should be interpreted as descriptive, observational results rather than definitive comparative conclusions. Differences in institutional treatment strategies may also have affected results. Larger, prospective studies are needed.

## Conclusion

We report outcomes of CSF with concomitant olecranon fracture. Postoperative results depended on CSF severity. A posterolateral extended approach provided effective exposure to both anterior and posterior articular surfaces. Posterolateral plating for Dubberley 3B fractures may be associated with poorer outcomes. Despite a high complication rate, appropriate surgical strategies can achieve favorable functional recovery. However, given the small sample size and observational design, these findings should be regarded as preliminary and hypothesis-generating rather than definitive evidence favoring any particular fixation method.

## Declaration of generative AI and AI-assisted technologies in the writing process

During the preparation of this work the authors used Claude (Anthropic) in order to translate specific Japanese text segments into English. After using this tool/service, the authors reviewed and edited the content as needed and take full responsibility for the content of the publication.

## Disclaimers:

Funding: No funding was disclosed by the authors.

Conflicts of interest: The authors, their immediate families, and any research foundation with which they are affiliated have not received any financial payments or other benefits from any commercial entity related to the subject of this article.
